# Risk factors and the CCTA application in patients with vulnerable coronary plaque in type 2 diabetes: a retrospective study

**DOI:** 10.1186/s12872-024-03717-1

**Published:** 2024-02-04

**Authors:** Weihong He, Tingsong Fang, Xi Fu, Meiling Lao, Xiuyun Xiao

**Affiliations:** 1 Department of Radiology, Foshan Hospital of Traditional Chinese Medicine, Guangzhou University of Traditional Chinese Medicine, Foshan, China; 2 Department of Endocrinology, Foshan Hospital of Traditional Chinese Medicine， Guangzhou University of Traditional Chinese Medicine, Foshan, China

**Keywords:** Type 2 diabetes mellitus, Vulnerable coronary plaque, CT angiography, Intravascular ultrasound

## Abstract

**Background:**

Diabetes is an independent risk factor for cardiovascular disease. The purpose of this study was to identify the risk factors for vulnerable coronary plaques (VCPs), which are associated with adverse cardiovascular events, and to determine the value of coronary CT angiography (CCTA) in patients with type 2 diabetes mellitus (T2DM) and VCPs.

**Methods:**

Ninety-eight T2DM patients who underwent CCTA and intravascular ultrasound (IVUS) were retrospectively included and analyzed. The patients were grouped and analyzed according to the presence or absence of VCPs.

**Results:**

Among the patients with T2DM, time in range [TIR {the percentage of time blood glucose levels were in the target range}] (OR = 0.93, 95% CI = 0.89–0.96; *P* < 0.001) and the high-density lipoprotein-cholesterol (HDL-C) concentration (OR = 0.24, 95% CI = 0.09–0.63; *P* = 0.04) were correlated with a lower risk of VCP, but the triglycerides (TG) concentration was correlated with a higher risk of VCP (OR = 1.79, 95% CI = 1.01–3.18; *P* = 0.045). The area under the receiver operator characteristic curve (AUC) of TIR, and HDL-C and TG concentrations were 0.76, 0.73, and 0.65, respectively. The combined predicted AUC of TIR, and HDL-C and TG concentrations was 0.83 (*P* < 0.05). The CCTA sensitivity, specificity, false-negative, and false-positive values for the diagnosis of VCP were 95.74%, 94.12%, 4.26%, and 5.88%, respectively. The identification of VCP by CCTA was positively correlated with IVUS (intraclass correlation coefficient [ICC] = 0.90).

**Conclusions:**

The TIR and HDL-C concentration are related with lower risk of VCP and the TG concentration was related with higher risk of VCP in patients with T2DM. In clinical practice, TIR, HDL-C and TG need special attention in patients with T2DM. The ability of CCTA to identify VCP is highly related to IVUS findings.

**Supplementary Information:**

The online version contains supplementary material available at 10.1186/s12872-024-03717-1.

## Introduction

The prevalence of type 2 diabetes (T2DM) among adults in China was 11.2% in 2017, and most patients with T2DM were also at high risk for cardiovascular disease (CVD) according to the Guide for the Prevention and Treatment of T2DM in China (version 2020) [1]. Atherosclerotic cardiovascular disease (ASCVD) comprises a significant portion (81.7%) of the cardiovascular conditions associated with T2DM. Major adverse cardiovascular events brought on by ASCVD are at risk due to T2DM independently [2]. Thin fibrous caps (thickness < 65 μm) and large necrotic cores with spotty calcifications and positive heaviness are the pathologic characteristics of vulnerable plaque and thin-capped fibroatheromas. Major adverse cardiovascular events and thin-capped fibroatheroma plaque rupture are thought to be closely related [3, 4]. In lesions with 50% stenosis of the coronary artery diameter, approximately one-half of the vulnerable coronary plaque (VCP) will rupture [5]. Therefore, predicting VCPs and managing VCPs effectively is essential to preventing major adverse cardiovascular events.

Intravascular ultrasound (IVUS) and optical coherence tomography (OCT) are currently the main tools for vascular diagnosis and interventional surgery guidance. IVUS has higher tissue penetration, can evaluate the entire structure of the coronary artery, including the external elastic membrane, and can more truly reflect the overall status of coronary artery plaque [6]. OCT is able to reveal more detail than IVUS due to higher resolution; however, higher resolution comes at the cost of a lower penetration depth [7]. Due to invasiveness, there are limitations in the clinical follow-up management of VCPs. Coronary CT angiography (CCTA) is a popular non-invasive imaging examination technique for diagnosing coronary artery disease. The negative predictive value and sensitivity are high and the identification of VCPs has an area under the receiver operator curve (AUC) of 0.91 [8].

Recent research has demonstrated that the morphology and composition of plaque, including the “napkin sign,” low attenuation plaque, spotty calcifications, and positive remodeling, which identify VCPs, can be accurately assessed by CCTA. These markers also exhibit a strong correlation with IVUS (*r* = 0.928) [9].

Improved spatial and temporal resolution, a quicker scan mode, and full heart coverage with wide-detector or dual-source CT are some of the unique advantages of current generation scanners. Dual-source CT features an ultra-high-pitch mode scan mode that can photograph the heart in < 300 ms and a wide-detector CT can capture pictures of the entire heart in a single heartbeat [10]. CT may be used to accurately measure and quantify the volume of coronary plaque non-invasively [11]. By imaging perivascular plaque, flow, and fat, new cardiac CT methods may evaluate coronary artery inflammation, which may be a significant advance in determining the “residual risk” that is missed by plaque or ischemia imaging alone [12, 13].

### Objective

To effectively manage VCPs and lessen the negative clinical outcomes in T2DM patients with VCPs, the risk factors for VCP in patients with T2DM were examined in the current study, as well as the usefulness of CCTA in clinically monitoring VCPs in T2DM patients.

## Patients and methods

### Patient selection

According to the principles of the Declaration of Helsinki, this retrospective study was approved by the Ethics Committee of Foshan Hospital of Traditional Chinese Medicine,Guangzhou University of traditional Chinese Medicine (No. KY【2022】182). Written informed consent was waived by the Institutional Committee because this was a retrospective study.

Patients with T2DM who were hospitalized in the Endocrinology Department of our hospital from January 2019 to December 2021 were retrospectively studied. IVUS and CCTA examinations were performed on all patients given the efficiency in diagnosis of plaques and coronary artery disease [6, 8, 9, 14]. Gender, age, disease progression, body mass index (BMI), glycosylated hemoglobin (HbA1c), time in range (TIR [the percentage of time of blood glucose levels in a target range]), and other clinical information were included (Table 1). The diagnostic standard was the China Diabetes Prevention and Treatment Guidelines (2017), which follows the WHO diagnostic standards [15]. Consecutive T2DM patients who met the inclusion and exclusion criteria and had CCTA and IVUS examinations within 4 weeks were included in the research.

**Table 1:** Clinical characteristics of the enrolled subjects

Variables	Total (*n* = 98)	Non-VCP (*n* = 51)	VCP (*n* = 47)	*P*
Male, n(%)	57(54.30)	29(56.90)	28(59.60)	0.11
Age (years)	72.5(43,88)	68.5(43,88)	72(43,82)	0.62
Diabetes duration (years)	10(0.50,23)	11(0.50,20)	8(1,23)	0.93
BMI (kg/m^2^)	24.45(18.40,28.90)	24.68 ± 2.58	24.10(19.50,27.90)	0.55
SBP (mmHg)	135 ± 12	136(105,151)	137 ± 12	0.25
DBP (mmHg)	84(60,99)	80(62,99)	87(60,98)	0.04
HbA1c (%)	6.0 ± 1.1	4.8 ± 1.1	6.2 ± 1.0	0.04
HbA1c (mmol/mol)	42.1 ± 14.3	29.0 ± 12.9	44.3 ± 16.7	0.04
GLU (mmol/L)	7.0(4.8,13.8)	6.4(5.3,11.9)	7.9(5.3,13.8)	<0.001
TIR (%)	57.50(21,82)	67(23,82)	49.21 ± 15.03	<0.001
TBR (%)	0(0,33)	0(0,20)	0(0,33)	0.78
TAR (%)	39.23(6,78)	29(15,78)	45.43 ± 16.03	<0.001
SIRI	0.98(0.12,3.02)	0.66(0.23,2.96)	1.05(0.12,3.02)	0.001
CRP (mg/L)	5.22(2, 21.60)	3.07(0.20,21.60)	6.75(0.30,19.50)	0.13
TC (mmol/L)	4.9 ± 1.2	4.8 ± 1.2	5.1 ± 1.2	0.45
TG (mmol/L)	1.3(0.2,5.6)	0.9(0.2,4.3)	1.6(0.3,5.6)	0.01
LDL-C (mmol/L)	3.3(1.8,5.7)	3.0(1.8,5.7)	3.1(1.8,4.0)	0.42
HDL-C (mmol/L)	1.2(0.5,3.7)	1.3(0.8,2.1)	1.0(0.5,3. 7)	<0.001
Statin medication, n (%)	37(37.8)	18(35.3)	19(40.4)	0.60
Aspirin medication, n (%)	28(28.6)	13(25.5)	15(31.9)	0.48

Data are the mean ± standard deviation, quartile (min, max), or percentage unless otherwise stated. VCP: vulnerable coronary plaque; BMI: body mass index; HbA1c: glycosylated hemoglobin; GLU: glucose; TIR: time in range (the percentage of time of blood glucose levels in a target range); TBR: time below the target glucose range; TAR: time above the target glucose range; SIRI: systemic inflammatory response index; CRP: C-reactive protein; TC: total cholesterol; TG: triglycerides; LDL-C: low-density lipoprotein-cholesterol; HDL-C: high-density lipoprotein-cholesterol

The inclusion criteria were as follows: age > 18 years; a T2DM diagnosis; stability of the hypoglycemic regimen during the first 3 months; and availability of pertinent CCTA and TIR data. The exclusion criteria were as follows: other forms of diabetes (type 1 or gestational diabetes mellitus); severe or recurrent hypoglycemic episodes within the previous 3 months; a history of malignancy or CVD; a mental disorder; or severe kidney or liver dysfunction.

### Anthropometric and biochemical measurements

Anthropometric measures were collected by the same group of nurses and laboratory technicians. Height, weight, and systolic and diastolic blood pressure were collected. Height squared (m^2^) divided by weight (kg) was used to determine the BMI (kg/m^2^). Three blood pressure readings were taken with a standard mercury sphygmomanometer after sitting quietly for at least 5 min and the results were averaged. After a 10-h overnight fast the day before the continuous glucose monitoring (CGM) assessment, venous blood samples were obtained at 6:00 am. The systemic inflammatory response index (SIRI) was derived using the neutrophil (n), monocyte (m), and lymphocyte (L) counts in peripheral blood (SIRI = n×m/L). An Olympus AU5400 (city, Japan) biochemical analyzer was used to determine triglycerides (TG), total cholesterol (TC), high-density lipoprotein-cholesterol (HDL-C), and low-density lipoprotein-cholesterol (LDL-C) concentrations. The glucose oxidase method was used to measure the fasting plasma glucose levels. High-performance liquid chromatography was used to measure HbA1c with a Variant II Hemoglobin A1c analyzer (TOSOH TSKgel G8, city, Japan).

### Intravascular ultrasound image acquisition

IVUS pictures were obtained with a 30- or 40-MHz imaging catheter (UltraCross or Atlantis 2.9Fr; Boston Scientific, Boston, MA, USA) by the same radiologist. The images were initially recorded in the medical DICOM format for recording IVUS images onto CDs or other digital media for offline examination. The IVUS images were then evaluated independently by two experienced physicians who were not aware of the patients’ clinical information or CCTA images. Plaque characteristics, including the presence or absence of a lipid pool, and soft, hard, and calcified plaque, were studied.

### CT parameters

CCTA imaging was performed by the same radiologist on a GE 256 revolution CT scanner (GE Healthcare,city, state, USA)or a Philips Brilliance 64-row spiral CT scanner (Philips Healthcare, city, The Netherlands). Prospective ECG gating was routinely performed. An artificial intelligence triggered scanning system was used to enhance the scan. The region of interest (ROI) was placed in the descending aorta 1 cm below the tracheal crest. The trigger threshold was set to 120 Hounsfield units (Hu). When the density reached the preset value, a cardiac volume scan was automatically triggered. Iopamidol (370 mg of I/mL), a non-ionic contrast agent, was injected via an elbow vein using a double-tube high-pressure syringe at a flow rate of 5.0 mL/s and a total volume of 50–80 mL.

### Qualitative analysis of VCP in coronary arteries by CCTA

Plaques showing positive remodeling, hypodense plaques, spotty calcifications, and the “napkin ring” sign on CCTA images were defined as VCPs [16]. All CCTA images used to diagnose VCPs were reviewed blindly by two senior associate physicians and discussed to determine if there were inconsistencies.

### Continuous glucose monitoring (CGM) parameters

Using a retrospective CGM system (Medtronic Inc., Northridge, CA, USA), subcutaneous interstitial glucose monitoring was carried out on 3 consecutive days for each patient, as previously described [17]. TIR was defined as the proportion of time spent throughout a 24-h period in the target glucose range of 3.9–10.0 mmol/L. Additionally, the duration of the blood glucose level falling below and rising above the target glucose range (TBR and TAR, respectively) were determined.

### Statistical analysis

Statistical analyses was performed using SPSS software (version 22; IBM Corp., Armonk, NY, USA). The chi-square test was used for categorical variables. A t-test and Mann Whitney U-test were used for continuous variables. Continuous variables were tested for normality and are presented as the mean +/- standard deviation if normally distributed and the median [interquartile range] if non-normally distributed. The intraclass correlation coefficient (ICC) is one of the most widely adopted reliability indices based on the analysis of variance (ANOVA) in the medical literature [18]. The ICC between CCTA and IVUS was determined using IVUS as the gold standard, and a value > 0.8 indicated good or outstanding reliability of the approach. Receiver operator characteristic curves were utilized to assess the predictive value of the risk factors and binary logistic stepwise regression was performed to determine the risk factors for VCP development in patients with T2DM. A P-value < 0.05 was considered statistically significant.

## Results

### Participants

**Fig. 1:**
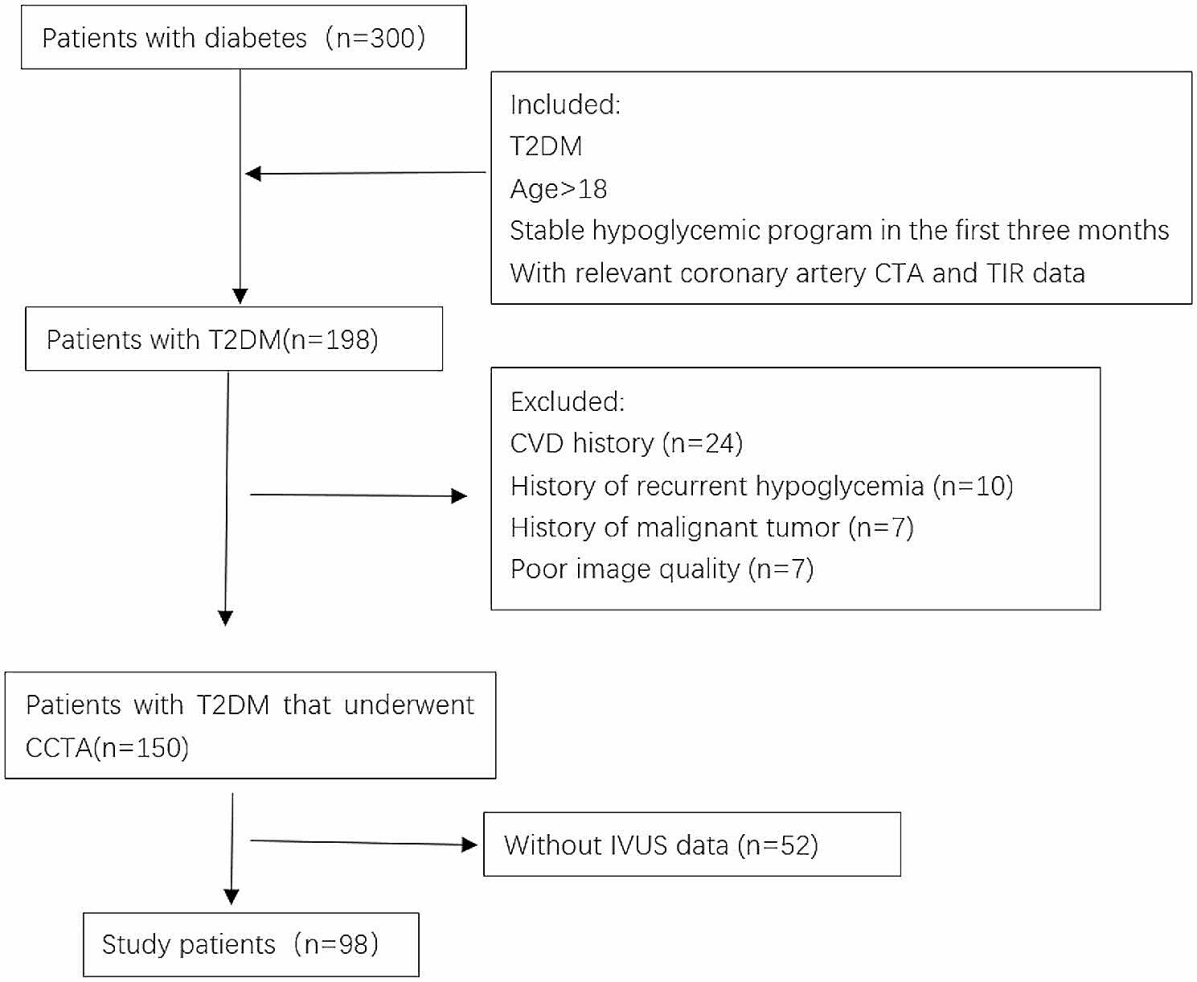
Flowchart showing study design

Based on the inclusion criteria, we studied 300 T2DM patients. Patients with a CVD history (*n* = 24), recurrent hypoglycemia history (*n* = 10), history of malignancy (*n* = 7), poor image quality (*n* = 7), and lacking IVUS data (*n* = 52) were excluded. A total of 198 T2DM patients were included in the final analysis (Fig. 1).

### Descriptive data

The clinical characteristics of the enrolled subjects are presented in Table 1. VCPs were detected in 47 (47/98) patients. Patients with VCPs had higher diastolic blood pressure (DBP), glucose (GLU), HbA1c, TAR, SIRI, and TG, and a lower TIR and HDL-C compared to the patients without VCPs (all *P* < 0.05).

### Binary logistic stepwise regression analysis on the risk factors for VCP in T2DM patients

The binary logistic stepwise regression analysis is shown in Table 2. The analysis showed that the TG concentration is related with higher risk for VCP in patients with T2DM (OR = 1.79, 95% CI = 1.01–3.18; *P* = 0.045). The higher the TG concentration, the higher the risk of VCP. The TIR (OR = 0.93, 95% CI = 0.89–0.96; *P* < 0.001) and HDL-C concentration (OR = 0.24, 95% CI = 0.09–0.63; *P* = 0.04) were associated with a lower risk of VCP.

**Table 2:** Logistic regression analysis of related factors for VCP in T2DM

Factors	B	SE	Wald	*P*	OR	95% CI
TIR	-0.08	0.02	16.54	<0.001	0.93	0.89–0.96
TG	0.58	0.29	4.02	0.045	1.79	1.01–3.18
HDL-C	-1.43	0.49	8.45	0.04	0.24	0.09–0.63
Constant	5.36	1.50	12.75	<0.001	213.41	-

TIR: time in range (the percentage of time of blood glucose levels in a target range); VCP: vulnerable coronary plaque; TG: triglycerides; HDL-C: high-density lipoprotein-cholesterol

**Fig. 2:**
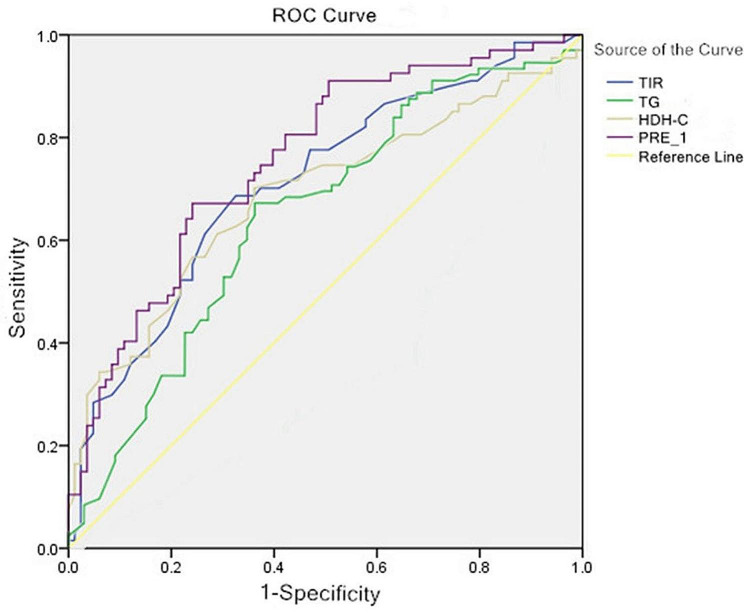
Receiver operator characteristic curve of time in range (the percentage of time of blood glucose levels in a target range), high-density lipoprotein-cholesterol, triglycerides, and probability for predicting VCP in T2DM.

The TIR, and TG and HDL-C concentrations, and various combinations were used to calculate the AUC (Fig. 2). The sensitivity for the TIR, and TG and HDL-C concentrations, and various combination predictions was 66.00%, 68.10%, 59.60%, and 66.00%, respectively. The specificity for the TIR, and TG and HDL-C concentrations, and various combination predictions was 80.40%, 62.70%, 82.40%, and 90.02% with AUC values of 0.76, 0.65, 0.73, and 0.83, respectively. The combined AUC threshold was 0.59 with a 66.00% sensitivity and a 92.02% specificity.

**Fig. 3:**
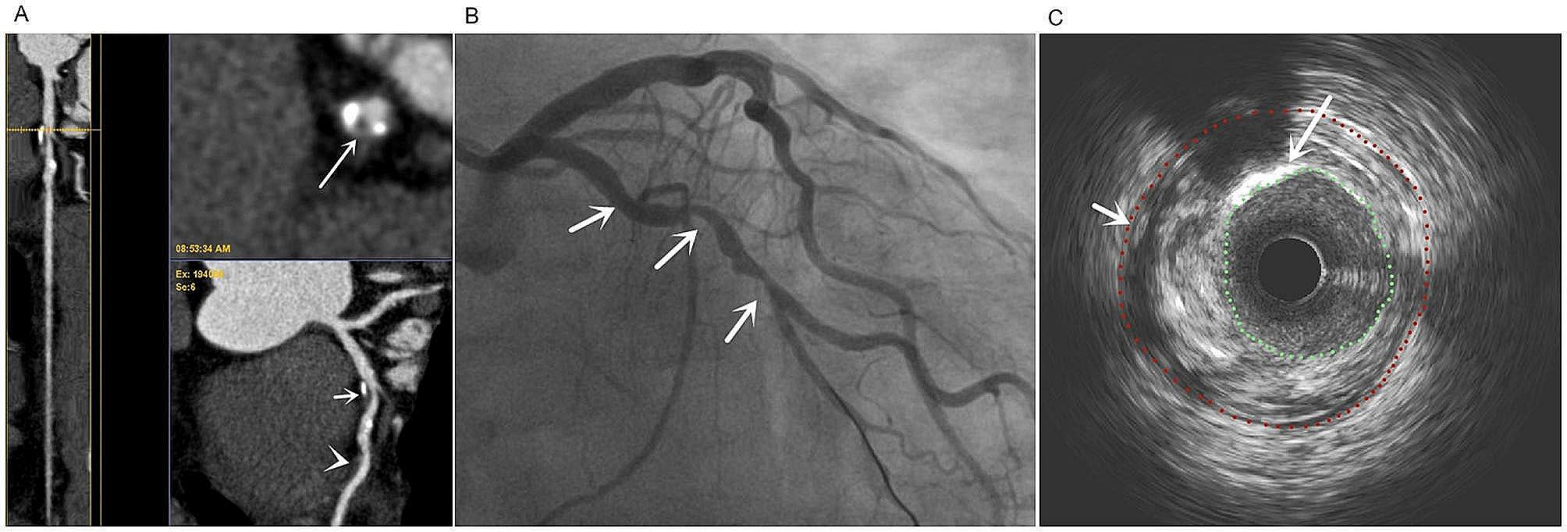
Representative image from a 67-year-old man diagnosed with T2DM for 8 years (TIR = 50%). **a**: CCTA showing multiple mixed plaques (short arrows) and non-calcified plaques (arrows) in LAD, and the transverse section of lumen showing a napkin sign (long arrow), indicating vulnerable plaque, **b**: DSA showing multiple stenosis of LAD lumen (arrow); **c**: IVUS showed levels corresponding to the CCTA napkin sign, mixed plaques in the LAD wall with high echogenicity of spotty calcifications (long arrow), and low echogenicity of lipid plaques (short arrow). Notes: TIR: time in range (the percentage of time of blood glucose levels in a target range); LAD: left anterior descending coronary artery

**Fig. 4:**
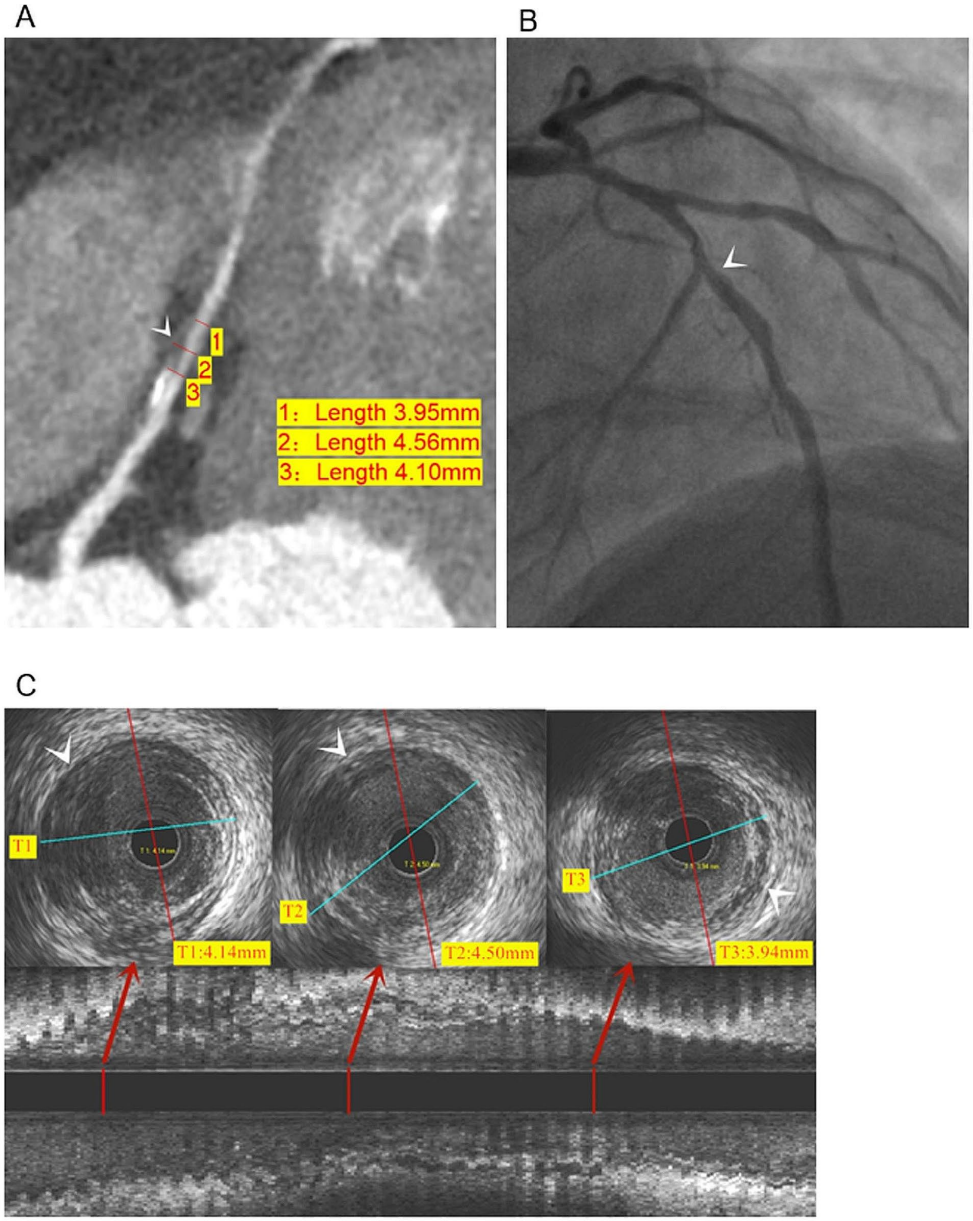
Representative image from a 53-year-old woman diagnosed with T2DM for 4 years (TIR = 35%, TG: 2.8 mmol/L, HDL-C: 1.1 mmol/L). **a**: CCTA showing multiple mixed plaques (arrows) and positive remodeling of the LAD branch, the positive remodeling index (PRI) was 0.13. **b**: DSA showed moderate stenosis of the local lumen of the LAD branch (arrows). **c**: IVUS showed low echogenicity of lipid plaques(arrows)and positive remodeling of the LAD branch; the PRI was 0.11. Notes: TIR: time in range (the percentage of time of blood glucose levels in a target range); TG: triglycerides; HDL-C: high-density lipoprotein-cholesterol; LAD: left anterior descending coronary artery

Table 3 shows the CCTA accuracy in diagnosing VCP. The sensitivity, specificity, false negative rate (FNR), and false positive rate (FPR) of CCTA in diagnosing VCP were 95.74%, 94.12%, 4.26%, and 5.88%, respectively. VCP detection by CCTA was significantly associated with IVUS (ICC = 0.90; Figs. 3 and 4).

**Table 3:** Accuracy of CCTA in diagnosing VCP

VCP		IVUS (n)		Total
		+	-	
CCTA (n)	+	45	3	48
	-	2	48	50
Total		47	51	98

VCP: vulnerable coronary plaque

## Discussion

The results of the current study showed that the TIR and HDL-C concentration are correlated with a lower risk of VCP, and the TG concentration is related with a higher risk of VCP in patients with T2DM. The ability of CCTA to identify VCP is highly related to IVUS findings.

### Pathologic basis of the clinical index reaction of T2DM VCP

ASCVD is a progressive arterial wall disease. The pathologic mechanism underlying ASCVD include inflammation, vascular lipid deposition, vascular plasticity, vascular fibrosis, and thrombosis [19]. A variety of physiologic changes induced by dyslipidemia, hyperglycemia, and insulin resistance include the development of atherogenic low-density lipoprotein (LDL), advanced glycation end products, and pro-inflammatory signaling, which affect various cell types in the arterial wall and lead to the development of atherosclerotic lesions [20]. The univariate analysis in this study confirmed that the formation of VCPs in patients with T2DM is related to the blood glucose level, inflammation, and blood lipid levels of patients. These findings are consistent with the pathologic process underlying ASCVD. VCPs were present in 47 of 98 diabetics in the current study, which is a higher rate (48.0%) than the rate (32.8%) reported by Huang et al. [21]. It has been shown that the prevalence of VCP in T2DM patients is much higher than non-diabetic populations [22]. Huang et al. think that the high rate is related with blood lipids and inflammation [21], Edsfeldt et al. think that T2DM atherosclerotic plaque is more likely to rupture and damage the repair response [22]. A recent study finds that patients with prediabetes, obesity and diabetes show more VCP phenotypes, and more extensive coronary atherosclerosis [23]. Furthermore, patients of a higher BMI is shown to have a greater extent of fibroatheroma, and glycemic status is an independent predictor of plaque erosion [23]. In our study, the T2DM patients had a commonly high BMI of over 24, and the HbA1c levels were significantly higher in VCP group than non-VCP. These two factors might lead to a higher VCP rate in our study than previous report, but the underlying reason may need more confirmation with a large-sample study. Also, the common feature of VCP is a thin fiber capsule (< 65 μm), a large amount of lipid, and increased macrophage activity [24]. Compared with non-diabetic patients, thin cap fibrous plaques are more common in diabetic patients (72% vs. 45%; *p* = 0.012) [25].

The formation of VCPs in patients with T2DM is a chronic pathologic process involving multiple factors, and the clinical factors often affect each other. Therefore, further binary logistic stepwise regression results showed that the TG concentration is related with higher risk of VCPs in patients with T2DM, and the TIR and HDL-C concentration are related with lower risk of VCPs. The TIR has been linked to T2DM complications and can provide blood glucose information that HbA1c cannot provide [26]. An increased TIR indicates that glycemic control is stable and patients spend less time hyperglycemic and/or hypoglycemic. As a result, patients with T2DM and a higher TIR have a lower incidence of VCPs. The current study also showed that the TAR difference between the presence and absence of VCPs in patients with T2DM is statistically significant, but not the TBR difference. The reason for this finding could be that the frequency of hyperglycemia in diabetic patients is significantly higher than hypoglycemia, indicating a non-normal distribution. The TBR usually has a much smaller impact on the TIR than the TAR. Glycemic control alone cannot completely prevent cardiovascular complications of diabetes. Multiple variables, including dyslipidemia, have been shown to contribute to the cardiovascular consequences of diabetes [27]. Diabetic lipids have complex intrinsic mechanisms (mainly elevated TG and reduced HDL-C concentrations). Hypertriglyceridemia may be central to the progression of CVD in diabetics. The TG concentration is elevated and lipoprotein oxidation and lipoproteinase activity are increased in diabetic patients, especially diabetics with poor glycemic control, resulting in an increase in oxidized protein components and small, LDL-C, which are involved in atherosclerosis [28]. Therefore, the TIR, and HDL-C and TG concentrations accurately indicate the risk of coronary VCP formation in T2DM patients, and improve clinical vigilance against VCP and related clinical events.

### Clinical management and monitoring of VCPs in T2DM patients

IVUS and OCT are the main intracoronary imaging (ICI) techniques, both of which have advantages and limitations. Moreover, there are invasive inspection or treatment technologies that are expensive and infrequently used in clinical practice that are not successful in detecting and controlling VCP [29]. CCTA not only accurately assesses coronary stenosis, but CCTA also detects plaque and identifies VCPs. Recent findings suggest that CCTA is the principal diagnostic technique for non-invasive examination of atherosclerosis with similar accuracy to IVUS beyond morphologic identification of the obstructed lumen [30–33]. The plaque volume assessed by CCTA was significantly associated (*r* = 0.98; *p* < 0.001) with the IVUS measurements of 76 plaques [34].

The present study showed that CCTA identification of VCPs was positively correlated with IVUS, which is similar to that reported in the literature [9]. CCTA characteristics of VCP reflect the histopathologic characteristics, including a thin fiber cap (< 65 μm), a large lipid necrotic nucleus (> 10% of the plaque area), positive vascular remodeling, plaque hemorrhage, and inflammatory infiltration [35]. These high-risk characteristics have been linked to poor clinical outcomes or significant hemodynamic significance [36]. According to studies, coronary atherosclerotic plaques in T2DM patients have a larger average necrotic core and a higher total plaque load when compared to non-diabetic patients [35]. The majority of plaques that resulted in late clinical events exhibited at least one VCP on the baseline CTA, and in 54% of instances, two VCP signals according to a study on predictors of late plaque events in asymptomatic T2DM [37]. However, a considerable proportion of patients with myocardial infarcts and non-obstructed coronary arteries (i.e., < 50% coronary stenosis) are undertreated even though the 1-year myocardial infarction and death risk are comparable to patients with coronary artery disease [38]. This finding suggests that the risk of ASCVD is linked to atherosclerotic plaque as well as coronary stenosis. The quantitative assessment of VCP using CCTA can aid in the risk stratification of coronary artery disease in addition to predicting VCP and unfavorable cardiovascular events [39]. As a result, it is important to forecast and detect VCP in diabetics as early as possible. CCTA can quickly estimate plaque volume and stenosis severity that closely match IVUS and have prognostic value for an incipient myocardial infarction according to an international multicenter study [40].

This study showed that clinical indicators, such as the TIR, and HDL-C and TG concentrations, can effectively predict VCP based on a CCTA examination. The combined predicted AUC of the TIR, and HDL-C and TG concentrations was 0.83, with a sensitivity of 66.00% and a specificity of 90.02%. Therefore, the combination of clinical indicators (TIR, and HDL-C and TG concentrations) and CCTA is of great significance in the screening and monitoring of coronary artery disease in T2DM patients.

This study had certain limitations. First, this study was a retrospective study and cannot confirm the relationship between the progression of VCP and independent risk factors in patients with T2DM due to the impracticable multivariate regression analysis. Second, coronary plaque formation is a long process. The TIR was calculated from 3-day CGM data and it does not reflect the entire glycemic control history of patients. Moreover, the sample size of this study was small and the single-center data may have some impact on the results.

## Conclusion

The TIR and HDL-C concentration are related with lower risk of VCP and the TG concentration was related with higher risk of VCP in patients with T2DM. In clinical practice, TIR, HDL-C and TG need special attention in patients with T2DM. The ability of a CCTA examination to identify VCP is positively correlated with IVUS. The combination of clinical indicators (TIR, and HDL-C and TG concentrations) and CCTA examination can be used to further screen and monitor coronary heart disease in T2DM patients.

### Electronic supplementary material

Below is the link to the electronic supplementary material.

Supplementary Material 1

## Data Availability

The data related to this study can be provided by the corresponding author on reasonable request.
